# Catheter-Based Renal Sympathetic Denervation Significantly Inhibits Atrial Fibrillation Induced by Electrical Stimulation of the Left Stellate Ganglion and Rapid Atrial Pacing

**DOI:** 10.1371/journal.pone.0078218

**Published:** 2013-11-06

**Authors:** Yuemei Hou, Jialu Hu, Sunny S. Po, Huan Wang, Ling Zhang, Feng Zhang, Kun Wang, Qina Zhou

**Affiliations:** 1 Department of Cardiovascular Diseases, The 6th People’s Hospital affiliated to Shanghai Jiaotong University, Shanghai, China; 2 Department of Cardiovascular Diseases, Zhongshan Hospital Fudan University, Shanghai, China; 3 Heart Rhythm Institute and Department of Medicine, University of Oklahoma Health Sciences Center, Oklahoma City, Oklahoma, United States of America; 4 Arrhythmia Research Lab, First Affiliated Hospital of Xinjiang Medical University, Urumqi, China; University of Buenos Aires, Faculty of Medicine. Cardiovascular Pathophysiology Institute, Argentina

## Abstract

**Background:**

Sympathetic activity involves the pathogenesis of atrial fibrillation (AF). Renal sympathetic denervation (RSD) decreases sympathetic renal afferent nerve activity, leading to decreased central sympathetic drive. The aim of this study was to identify the effects of RSD on AF inducibility induced by hyper-sympathetic activity in a canine model.

**Methods:**

To establish a hyper-sympathetic tone canine model of AF, sixteen dogs were subjected to stimulation of left stellate ganglion (LSG) and rapid atrial pacing (RAP) for 3 hours. Then animals in the RSD group (n = 8) underwent radiofrequency ablation of the renal sympathetic nerve. The control group (n = 8) underwent the same procedure except for ablation. AF inducibility, effective refractory period (ERP), ERP dispersion, heart rate variability and plasma norepinephrine levels were measured at baseline, after stimulation and after ablation.

**Results:**

LSG stimulation combined RAP significantly induced higher AF induction rate, shorter ERP, larger ERP dispersion at all sites examined and higher plasma norepinephrine levels (P<0.05 in all values), compared to baseline. The increased AF induction rate, shortened ERP, increased ERP dispersion and elevated plasma norepinephrine levels can be almost reversed by RSD, compared to the control group (P<0.05). LSG stimulation combined RAP markedly shortened RR-interval and standard deviation of all RR-intervals (SDNN), Low-frequency (LF), high-frequency (HF) and LF/HF ratio (P<0.05). These changes can be reversed by RSD, compared to the control group (P<0.05).

**Conclusions:**

RSD significantly reduced AF inducibility and reversed the atrial electrophysiological changes induced by hyper-sympathetic activity.

## Introduction

Atrial fibrillation (AF) is a complex arrhythmia with multiple mechanisms. Electrical, structural and neural remodelling contribute to the AF substrate. Sympathetic activity constitutes an important factor in the pathogenesis of AF [1]–[5]. Strategies aiming at reducing sympathetic activity potentially protect against the initiation and maintenance of AF. Ablation of bilateral stellate and thoracic sympathetic ganglia can significantly reduced paroxysmal atrial tachyarrhythmia episodes induced by sympathetic discharges in dogs [6]. Catheter-based renal sympathetic denervation (RSD) is an effective and interventional therapy for patients with resistant hypertension [7]–[10]. Recently, several studies have demonstrated that the efficacy of RSD on AF. Linz et al. [11] reported that RSD reduced blood pressure, atrial effective refractory period (ERP) shortening and inducibility of vagally mediated atrial fibrillation in a model of obstructive sleep apnea. Furthermore, they also showed that electrical baroreflex stimulation significantly shortened atrial refractoriness, thereby causing increased AF inducibility. In contrast, RSD did not significantly modulate atrial refractoriness or AFinducibility [12]. Zhao et al. [13] showed that RSD could decrease episodes of AF during short-time rapid atrial pacing, which might correlate with decreased activity of both renin–angiotensin–aldosterone system (RAAS) and renal sympathetic nerve. However, the AF models in those three studies were all associated with increased vagal activity. The effects of RSD on atrial electrophysiological and AF inducibility remains controversial and the mechanisms underlying the effects remains unclear. Whether RSD could reduce AF inducibility induced by hyper-sympathetic activity is unknown.

In the present study, we developed an animal model by delivering rapid atrial pacing in the presence of a hyper-sympathetic tone induced by stimulation of left stellate ganglion (LSG) to evaluate the effects of RSD on AF inducibility, atrial electrophysiological changes and cardiac autonomic activity.

## Materials and Methods

### Ethics Statement

This study was carried out in strict accordance with the recommendations in the Guide for the Care and Use of Laboratory Animals of the National Institutes of Health. The protocol was approved by the Institutional Animal Care and Use Committee of the First Affiliated Hospital of Xinjiang Medical University (Permit Number: IACUC-20110325009), and conformed to the guidelines of the Association for Assessment and Accreditation of Laboratory Care (AAALAC). All surgery was performed under sodium pentovarbital anesthesia, and all efforts were made to minimize suffering [14].

### Animal and Groups Setting

Sixteen adult mongrel dogs weighing 18 to 22 kg were anesthetized with sodium pentobarbital (20 mg/kg) and ventilated with room air by a positive pressure respirator. Ketamine (2 mg/kg) was used for induction of general anesthesia and analgesic. Core body temperature was maintained at 36.5±1.5°C. Standard ECG leads were continuously recorded to determine heart rate and rhythm. Sixteen dogs were randomly divided into two groups. The control group (n = 8) underwent rapid atrial pacing (RAP) and the left satellite ganglion (LSG) sitimulatioin for 3 hours but without renal sympathetic nerve ablation. The renal sympathetic denervation (RSD) group (n = 8) underwent RAP and LSG sitimulatioin for 3 hours and followed by catheter-based radiofrequency ablation of the renal sympathetic nerve.

### A Canine Model of Acute Atrial Fibrillation Induced by the Left Stellate Ganglion Electrical Stimulation Combined with Rapid Atrial Pacing

Electrical stimulation of the left stellate ganglion (LSG) combined rapid atrial pacing (RAP) for 3 hours was used to establish a canine model of acute AF mediated by hyper-sympathetic activity.

### The Electrical Stimulation of LSG

**Figure 1:**
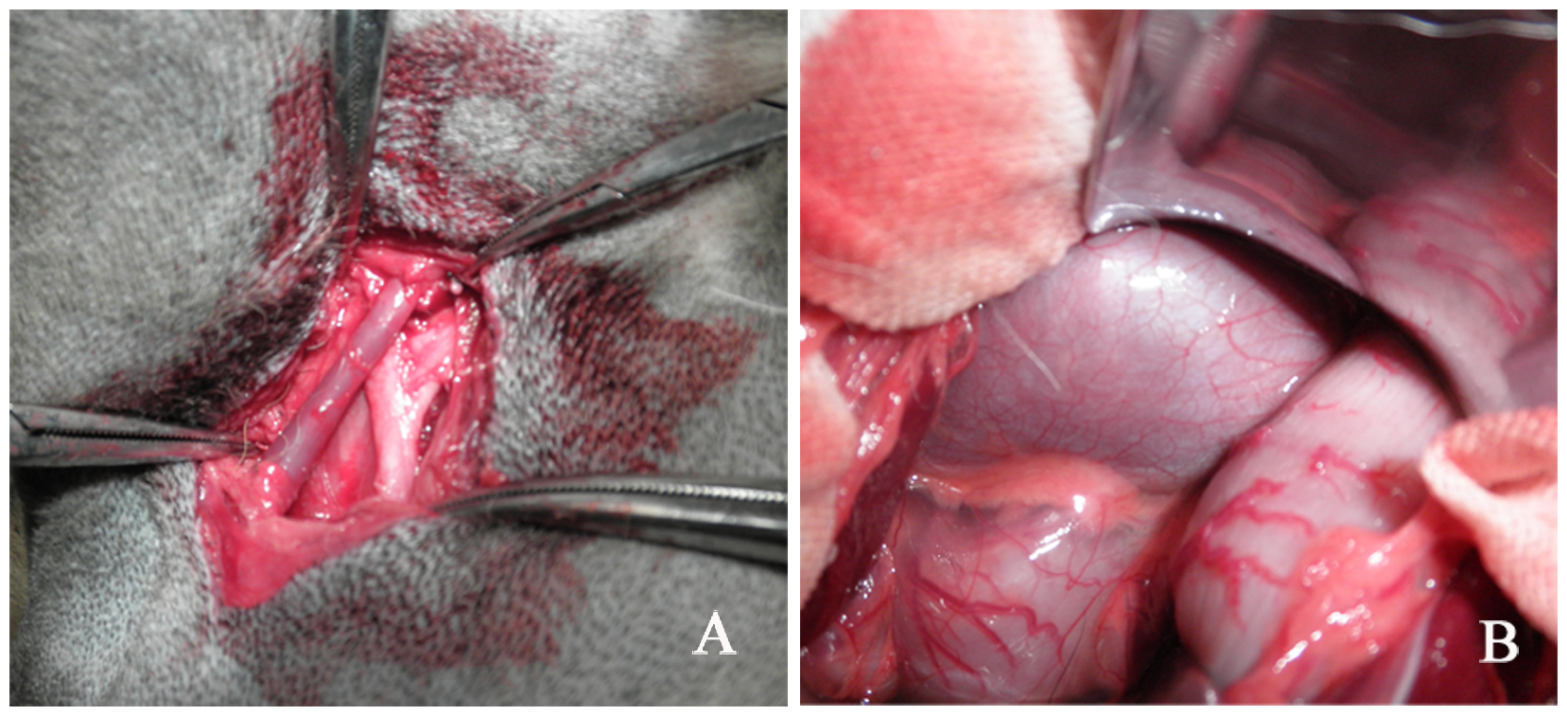
Representative images showing a stellate ganglion and renal hilum. A shows a star-shaped stellate ganglion behind the subclavian artery. B shows the renal hilum before dissection. The renal artery and renal sympathetic nerve enter the kidney through the renal hilum.

Anaesthesia was performed as described above. Vertical paramedian incision was made in the supraclavicular fossa. Behind the subclavian artery and vertebral artery, and in the adipose tissue in front of the seventh cervical vertebra, a star-shaped SG was visible on the left side (Figure 1A). The adipose tissue surrounding the SG was bluntly dissected with a glass dissecting needle to expose its branch and the cardiac sympathetic nerve. The LSG was then stimulated by GRASS S88 Nerve and Muscle Stimulator (Astro-Med Inc, USA) at a gradual level of 2 V to 10 V (20 Hz, 2-ms pulse width) for a period of 30 seconds. The stimulation threshold of SG is defined as the current required to produce a rise of 20% or more in systolic blood pressure (SBP) or heart rate [15], once the stimulation threshold was established, the LSG was then continuously stimulated (20 Hz, 2-ms pulse width, threshold voltage) for 3 hours.

### Rapid Atrial Pacing (RAP)

The right femoral arteries were used for recording of blood pressure (via a pressure transducer). The right external jugular vein was cannulated for catheter insertion into the right atrium (RA) to record right atrial potentials and to induce rapid atrial pacing. A left-sided thoracotomy was performed at the fourth intercostal space. Multi-electrode catheters were sutured to the left atrial appendage (LAA), left superior pulmonary vein (LSPV) and left inferior pulmonary vein (LIPV) to record pulmonary vein (PV) and atrial potentials. Continuous RAP in all the dogs was delivered (600 bpm, twice-threshold current, 0.5 ms in duration) at the right atrium (RA) for 3 hours.

### Measurement of AF Induction Rate

To calculate mean AF induction rate, AF was induced 6 times with S1S1 stimuli at 600 bpm (2 ms in duration, fourfold threshold current). AF induction rate was defined as (the relative ratio of successful induction frequency to total frequency of stimulation)×100%. AF was defined as irregular atrial rates >500 beats/min and a duration >5 seconds associated with irregular atrioventricular conduction [16]. AF lasting >30 minutes was considered sustained.

### Catheter -based Renal Sympathetic Denervation

After completion of all the above stimulation and measurement, laparotomy was performed through a midline incision. A peritoneal window was created to expose the renal hilum (Figure 1B). Steerable 6 F radiofrequency catheter (HL-75F, Sichuan, P. R. China) was introduced into the right and left renal artery from the femoral artery. The tip of the catheter was positioned under direct vision to make sure that it was accurately introduced into the renal artery on the both sides. In the control group, after the catheter was accurately introduced into the renal artery on the both sides, no further procedures were performed for 30 minutes (sham ablation). In the RSD group, we evaluated the effects of the renal sympathetic nerve by electrical stimulation (5 V, 20 Hz, 2-ms, Grass S88 nerve stimulator, USA) at the proximal renal artery for 30 seconds before and after bliateral RSD. Each discrete radiofrequency ablation (of approximately 6 to 8 watts) lasted 60 seconds, and total four ablations were performed both longitudinally and circumferentially within each renal artery. The left RSD was performed at first, followed by 4 ablations of the right RSD immediately. The electrical stimulation and ablation of the renal sympathetic nerve lasted for approximately 30 minutes.

### Electrophysiological Measurements

Electrophysiological measurements were made every one hour immediately after RAP and LSG stimulation and after cather-based RSD and sham ablation.

Electrophysiological recordings including effective refractory period (ERP) and ERP dispersion were measured before pacing and stimulation as baseline values, after pacing and stimulating for 3 hours (stimulation) and after ablation of LSG or bilateral renal sympathetic nerve. Total duration of reading was 10 minutes.

Programmed stimulation at the atrial and PV sites was performed using a cardiac programmable stimulator (Lead-2000 EP CONTROL, Sichuan, P. R. China). The ERP was defined as the longest S1S2 interval that failed to produce a response. It was measured at an atrial pacing cycle length of 300 ms and the S1–S2 intervals were decreased from 200 ms to refractoriness initially by decrements of 5 ms (S1:S2 = 8∶1, twice-threshold current, 0.5 ms in duration). ERP dispersion was defined as the coefficient of variation (standard deviation/mean) of the ERP at all 4 sites (LSPV, LIPV, LAA and RA).

### Analysis of Heart Rate Variability (HRV)

PowerLab (ML866/P, FE132 Bio Amp, ADInstruments, Australia) was used for ECG measurement. Three self-adhesive ECG electrodes are administered for recording. All signals were then analyzed using the HRV Module of LabChart \ro V7 software for HRV time domain analysis and frequency domain analysis.

HRV measurements included the following cardiovascular parameters: range of duration of RR-intervals (milliseconds, ms); SDNN: standard deviation of all RR-intervals(ms), which reflects overall variation in the heart beat series; pNN50: number of successive difference of intervals which differ by more than 50 ms, as a proportion of total beat cycles, which sensitively reflects the parasympathetic activity. High-frequency (HF) components (0.15–0.4 Hz) are driven mainly via parasympathetic innervation of the heart; Low-frequency (LF) components (0.04–0.15 Hz) are driven mainly by sympathetic innervation of the heart; and LF/HF ratio reflects sympathovagal balance.

### Measurement of Plasma Level of Norepinephrine

Blood samples were collected from the femoral artery into a tube containing EDTA, and immediately centrifuged at 3000 rpm for 10 min at 4°C, and then finally stored at −80°C till analysis.The plasma norepinephrine level was determined by high-performance liquid chromatography (HPLC) with YWG-C18 column and electrochemical detection (Waters 2465, Milford, MA) [17].

### Histology

Non-ablated renal ganglion in the segments which were dissected from the renal arteries with their perivascular tissue in the 8 control group dogs for histological control. After the completion of all the electrophysiological measurement after the ablation, the renal arteries with their perivascular tissues from the RSD group and control group were immediately dissected and fixed in formaldehyde. Two months later, hematoxylin-eosin (HE) and silver staining and immunohistochemical staining were performed.

Multiple tissue blocks were sampled from the sites of the renal artery with or without ablation. The sections were stained with routine staining for structural examination. The slides were also silver stained and immunostained with antibodies to tyrosine hydroxylase (TH) for the sympathetic nerves, according to methods described previously [18], [19]. The primary antibodies used in this study were sheep poly-clonal anti-TH (1∶50 dilution; Thermo Scientific, USA, MJ1473853,). The secondary antibody was polink-2 plus polymer HRP detection system (For Goat Primary Antibody, GBI, USA). The silver staining was used for staining nerve fibers and nerve endings [20]. Light microscopy was used to examine the sections.

### Statistical Analyses

Qualitative data were expressed as a ratio and measurement data were expressed as means with SD. One-Sample Kolmogorov-Smirnov Test was used to test the normality of the measurement data. ANOVA for repeated measures was used to compare the changes at baseline, after stimulation and ablation. LSD was used for Post Hoc multiple comparisons. The chi-square test was used to compare the AF induction rate. Values of P<0.05 were considered statistically significant.

## Results

### RSD Significantly Reduced Systolic Blood Pressure (SBP) but not Heart Rate (HR)

To examine effects of denervation on HR, we compared HR between stimulation and denervation, between the control group and RSD group. We found that LSG stimulation induced mild elevation of HR in both the control group and RSD group when compared with that without simulation, however, no statistical significance was seen (P>0.05). By contrast, RSD induced mild reduction of HR when compared with that in LSG stimulation condition and the control group(P>0.05).

LSG stimulation significantly elevated SBP by approximately 1-fold in both the control group and RSD group when compared with that without LSG stimulation (the control group: baseline: 142.17±25.45 mmHg verse stimulation: 165.68±34.59 mmHg, P<0.05; the RSD group: baseline: 139.02±33.41 mmHg verse stimulation: 167.36±29.31 mmHg, P<0.05; Table 1). Whereas, RSD significantly reduced the SBP in RSD group when compared with that in the LSG stimulation condition and control group [the control group (150.98±19.66) mmHg verse the RSD group (114.74±23.29) mmHg, P<0.05; within RSD group: stimulation (167.36±29.31) mmHg verse ablation (114.74±23.29) mmHg, P<0.05; Table 1).

**Table 1:** Effect of RSD on HR and SBP(mean+/−standard deviation).

	Control Group			RSD Group		
	Baseline	Stimulation	Ablation	Baseline	Stimulation	Ablation
HR(bpm)	158.43±39.72	163.82±43.04	160.54±38.61	159.90±31.94	167.22±41.06	153.24±36.72
SBP(mmHg)	142.17±25.45	165.68±34.59*	150.98±19.66	139.02±33.41	167.36±29.31*	114.74±23.29^#†^

Note: RSD = Renal sympathetic denervation, HR = Heart rate, SBP = Systolic blood pressure.

* P<0.05 indicated significance between stimulation and baseline conditions,

# P<0.05 indicated significance between ablation and stimulation conditions.

† P<0.05 indicated significance between the control group and RSD group.

### RSD Reduced AF Induction Rate

**Figure 2:**
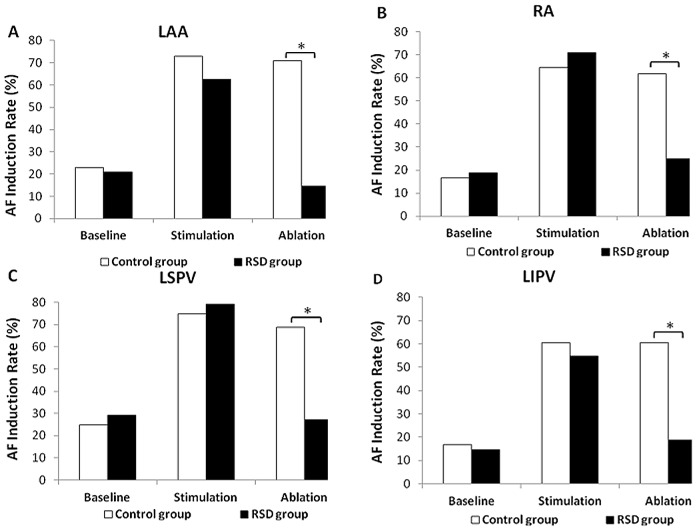
Changes in AF induction rate at RA, LA, LSPV and LIPV sites. RA = the right atrium, LA = the left atrium, LSPV = the left superior pulmonary vein, LIPV = the left inferior pulmonary vein, RSD = the renal sympathetic denervation. *P<0.05 indicated significant difference between the control group and the renal sympathetic denervation group.

When compared with those in the baseline condition, LSG stimulation with RAP significantly increased the AF induction rate at the LAA, RA, LSPV and LIPV sites by 49.98%, 47.92%, 50.00%, 43.75% in the control group, and by 41.67%, 52.08%, 50.04%, 40.09% in the RSD group, respectively. No significant difference in AF induction rate was found under baseline and after stimulation between the control group and RSD group. After ablation, RSD markedly decreased AF induction rate at the LAA, RA, LSPV and LIPV sites by 56.17%,36.83%, 41.84%, 41.92%, respectively compared to the control group (P<0.05), (Fig. 2).

### Effects of RSD on ERP

**Figure 3:**
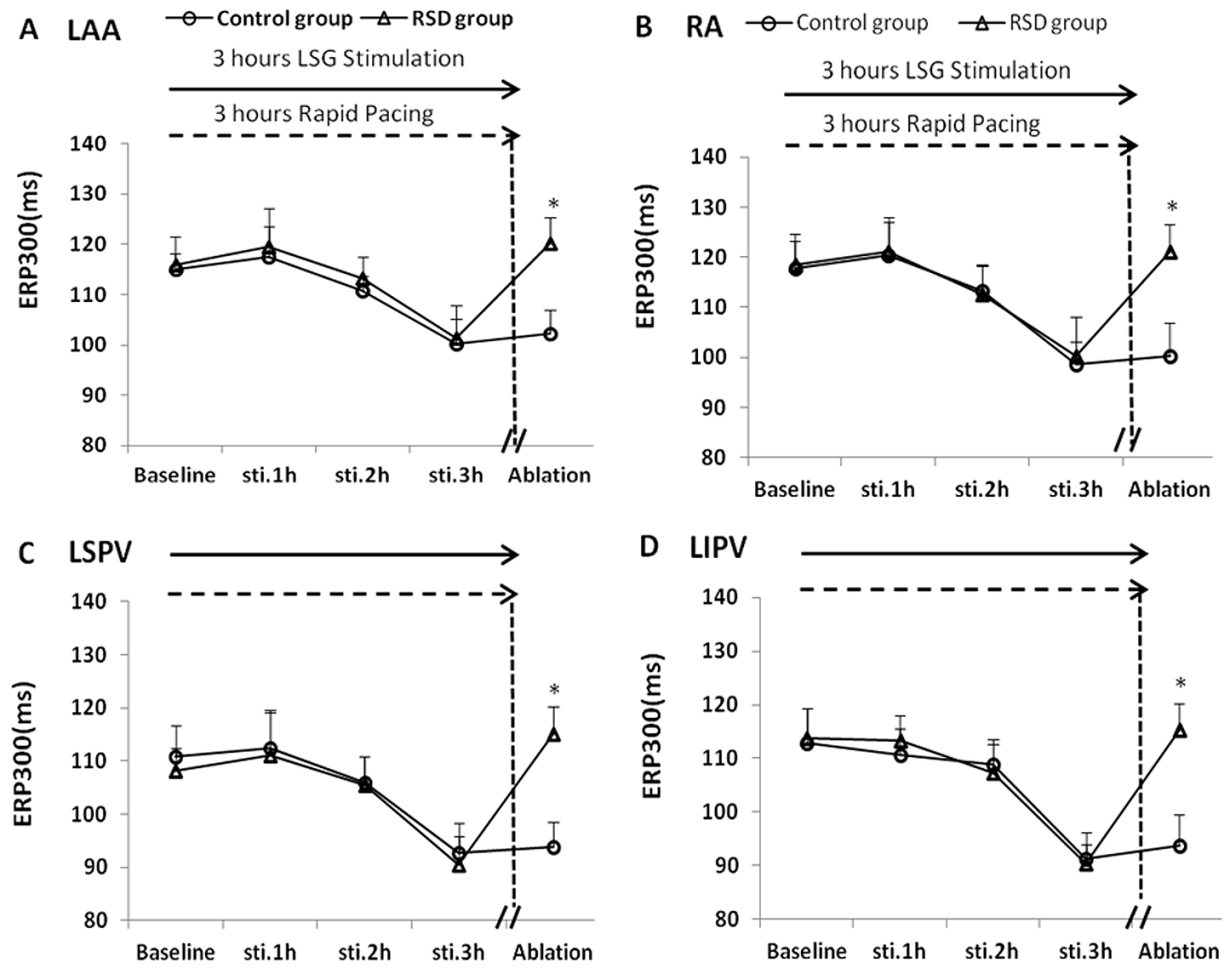
Changes in ERP at different sites. RA = the right atrium, LA = the left atrium, LSPV = the left superior pulmonary vein, LIPV = the left inferior pulmonary vein, RSD = the renal sympathetic denervation. *P<0.05 indicated significant difference between the control group and the renal sympathetic denervation group.

When compared with that at the respective baselines, LSG stimulation with RAP in 3 hours induced a pronounced ERP shortening at the LAA, RA, LSPV and LIPV sites by 14.67 ms, 19.17 ms, 18.14 ms, 21.52 ms in the control group, respectively (Fig. 3; P<0.05), and by 14.61 ms, 18.11 ms, 17.86 ms, 23.31 ms in the RSD group, respectively (Fig. 3; P<0.05). No significant difference in ERP was found at each site between the control group and RSD group under baseline and stimulation conditions. However, the ERP shortening can be reversed by RSD in the RSD group, when compared with that in the control group (Fig. 3; P<0.05).

### RSD Reduced ERP Dispersion

**Figure 4:**
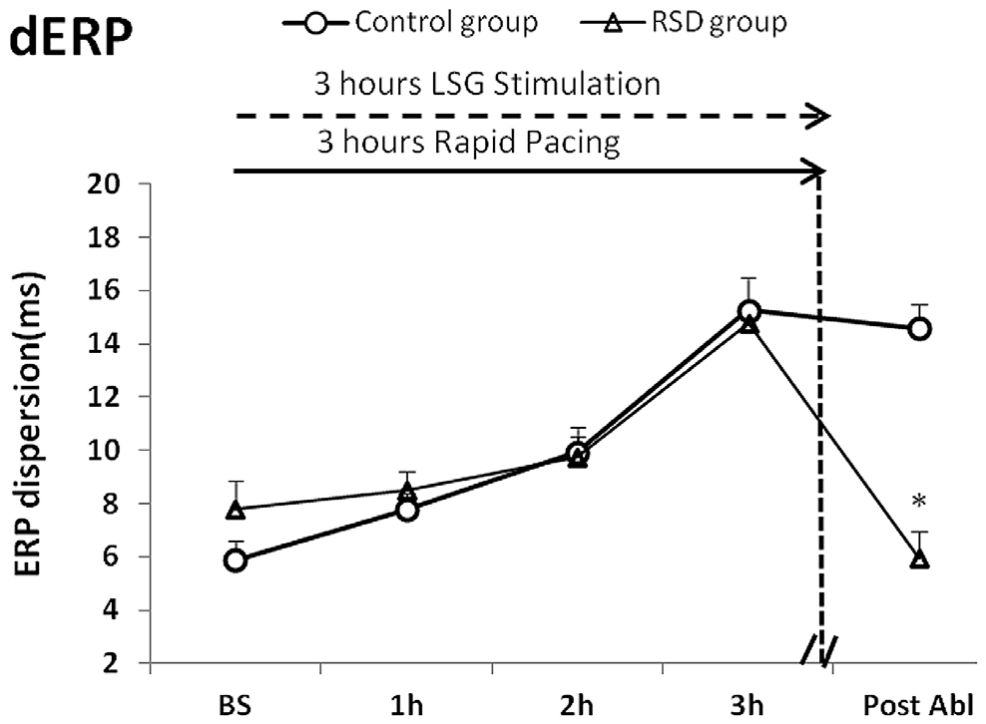
Changes in ERP dispersion (dERP) within different sites. RA = the right atrium, LA = the left atrium, LSPV = the left superior pulmonary vein, LIPV = the left inferior pulmonary vein, RSD = the renal sympathetic denervation. *P<0.05 indicated significant difference between the control group and the renal sympathetic denervation group.

When compared with that of the respective baselines, LSG stimulation with RAP in 3 hrs markedly increased ERP dispersion within the 4 sites in both the control group and RSD group [(the control group: baseline: 5.89 ms verse stimulation 15.25 ms, P<0.05; the RSD group: baseline: 7.80 ms verse stimulation 14.78 ms, P<0.05); Fig. 4]. No significant difference in ERP dispersion was found between the control group and RSD group under baseline and stimulation conditions. An increase in ERP dispersion can be reversed by RSD in the RSD group, compared with that in the control group [14.58 ms verse 5.97 ms, P<0.05); Fig. 4].

### Effects of RSD on Time and Frequency Domain HRV Parameters

Time domain parameters: When compared with the respective baseline, the RR-interval, SDNN and pNN50 were significantly shortened by LSG stimulation with RAP for 3 hours respectively by 27.6%, 55.8%, 60.8% in the control group, and by 31.7%, 48.7%, 53.3% in the RSD group, respectively (P<0.05, Table 2). These shortening can be completely reversed by RSD in the RSD group, when compared with those under LSG stimulation conditions and compared with those in the control group (P<0.05, Table 2).

**Table 2:** Effects of RSD on time and frequency domain HRV parameters (mean+/−standard deviation).

	Control Group			RSD Group		
	Baseline	Stimulation	Ablation	Baseline	Stimulation	Ablation
RR-Interval(ms)	364.25±69.35	263.27±44.05*	284.74±37.82	359.28±40.56	244.51±25.40*	380.24±69.47^#†^
SDNN(ms)	94.31±11.19	41.68±9.93*	43.57±7.30	98.36±7.98	50.41±8.55*	106.34±10.02^#†^
pNN50	34.48±4.58	13.51±3.06*	10.46±4.81	33.30±3.19	15.54±4.02*	25.51±2.93^#†^
LF(ms^2^)	253.56±106.52	871.70±233.28*	790.78±101.49	261.52±97.71	855.28±311.06*	290.78±221.49^#†^
HF(ms^2^)	408.62±179.01	967.09±363.51*	933.02±172.63	435.73±173.55	1017.54±431.32*	410.02±272.63^#†^
LF/HF	0.62±0.38	0.90±0.41*	0.85±0.15	0.60±0.47	0.84±0.35*	0.60±0.29^#†^

Note: RSD = Renal sympathetic denervation.

* P<0.05 indicated significant difference between stimulation and baseline conditions,

# P<0.05 indicated significant difference between ablation and stimulation conditions,

† P<0.05 indicated significance between the control group and RSD group.

Frequency domain parameters: When compared with the respective baseline, the LF, HF and LF/HF ratio were significantly increased by LSG stimulation with RAP for 3 hours by 2.43-, 1.39-, 0.45-fold in the control group, and by 2.27-, 1.34-, 0.40- fold in the RSD group, respectively (P<0.05, Table 2). These increase induced by LSG stimulation was completely reversed by RSD in the RSD group, when compared with those in the control group (P<0.05, Table 2).

### Plasma Norepinephrine Level

**Figure 5:**
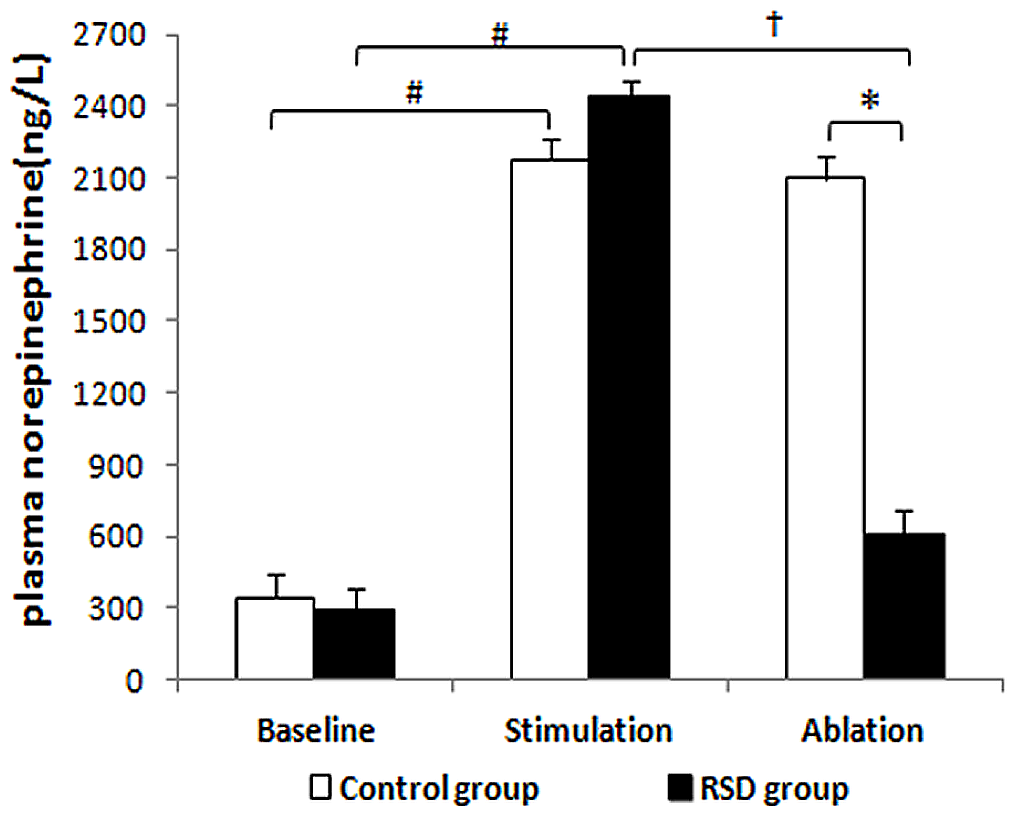
Plasma norepinephrine level. RSD = the renal sympathetic denervation. ^#^P<0.001 indicated significant difference between stimulation and baseline conditions. ^†^P<0.001 indicated significant difference between ablation and stimulation conditions. *P<0.001 indicated significant difference between the control group and the renal sympathetic denervation group.

The plasma norepinephrine levels were significantly elevated by LSG stimulation with RAP for 3 hours, by 6.38 -fold in the control group and by 8.4-fold in the RSD group, respectively (P<0.001, Fig. 5). No significant difference in the plasma norepinephrine level was found between the control group and RSD group under baseline and stimulation conditions. The elevated plasma norepinephrine levels were significantly reduced by RSD in the RSD group, when compared with that in the control group [(2099.51±94.31)ng/L verse (605.34±99.23)ng/L, P<0.001); Fig. 5].

### Histological Studies

**Figure 6:**
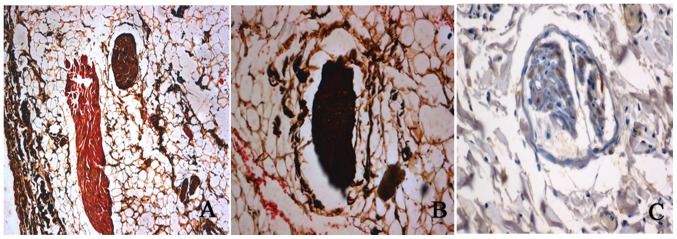
Sliver staining and tyrosine hydroxylase staining of the renal sympathetic nerves. A and B shows an example of silver staining of the renal artery nerves without ablation. C shows an example of tyrosine hydroxylase -positive staining of renal sympathetic nerves without ablation. Brown structures indicate positively stained nerve structures.

To verify whether radiofrequency ablation of the renal sympathetic nerve was successfully made, we performed HE staining and silver staining on the renal arteries. Silver staining of the nerve innervating the renal artery without ablation in the control group showed the nerve fiber distribution and normal structure of ganglionic cells, (Fig. 6A, 6B), and immunostaining results showed that TH-positive staining of renal sympathetic nerves without ablation (Fig. 6C). Brown structures indicated positively stained nerve structures. Intact (unablated) ganglion cells were observed in the middle of the section.

**Figure 7:**
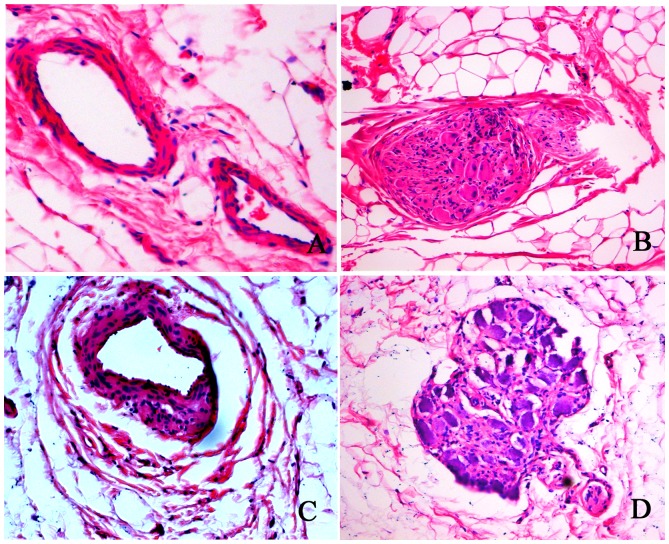
HE staining of the renal artery and renal nerves structures with or without ablation. (A) Non-ablated renal artery in control group. (B) Non-ablated renal ganglion in control group. (C) The ablated renal artery tissue was replaced by dense scar tissue that stained purple. (D) In the ablated renal artery, the ganglioinic cells become contracted morphologically and vacuolar degeneration was observed.

To compare effects of ablation on histological structures of the renal artery and renal nerves, we performed HE staining in the sections of the renal artery of the rats with or without ablation. HE staining of cells was uniformly arranged around the wall of normal renal arteries without ablation in the control group (Fig. 7A). However, after ablation in the RSD group, the ablated renal artery tissue was replaced by dense scar tissue that stained purple and it seemed that base membrane of the artery wall was disrupted and disorganized and become loose (Fig. 7C). In addition, the ganglionic cells after the ablation morphologically became contracted and characterized by vacuolar degeneration (Fig. 7D), when compared with that in non-ablated renal ganglion in the control group (Fig. 7B).

## Discussion

### Major Findings

In the present study, AF inducibility was facilitated and atrial electrophysiological changes (shortening of ERP, increasing of ERP dispersion) were enhanced by LSG stimulation and RAP. Cardiac hyper-sympathetic activity was manifested by shortened RR-interval, increased LF, HF and LF/HF ratio and elevated plasma norepinephrine levels after LSG stimulation. RSD reversed the altered cardiac sympathetic activity as well as AF inducibility and electrophysiological changes caused by LSG stimulation and RAP.

### The Role of Sympathetic Nerve Activity in AF and Acute Atrial Electrophysiological Changes

Previous studies have provided evidence that sympathetic nerve activity involved in the initiation and/or maintenance of AF. Infusion of isoprenaline (isoproterenol, ISO), a β-receptor agonist, induced AF in 5% of patients without history of AF and in 84% of patients with paroxysmal AF in a dose-dependent manner [3]. Several β-blockers have been shown to be effective in suppression of AF in patients with various heart diseases [21]. Chronic RAP increases the innervation of the atrial sympathetic nervous system [22], [23]. Furthermore, atrial sympathetic hyper-innervation was associated with chronic AF in patients [24]. Swissa and Tan et al. have reported that sympathetic hyper-innervation induced either by electrical stimulation of SG or nerve growth factor infusion can induce atrial nerve sprouting and a significantly higher incidence of paroxysmal AF and paroxysmal atrial tachycardia in dogs [15], [25]. By contrast, reduction of cardiac sympathetic outflow by cryoablation of the bilateral stellate ganglia and T2–T4 thoracic ganglia can effectively eliminate both paroxysmal atrial tachyarrhythmia in dogs with pacing-induced heart failure [26].

A recent study from our institute showed that LSG stimulation facilitated AF inducibility and aggravated atrial electrical atrial electrophysiological changes in the first 2 to 4 hours [27]. The inhibition of sympathetic nerve activity by left stellate ganglionectomy can reduce the AF induction and reverses the process of atrial electrophysiological changes. Therefore, in the present study, we used this acute AF model induced by LSG stimulation combined with RAP for 3 hours to observe the effects of RSD on AF inducibility and cardiac autonomic nerve activity. The indirect effect of LSG stimulation or sympathetic denervation on sympathetic activity was reflected by the changes of HR and significant SBP, HRV parameters and plasma norepinephrine levels. Cardiac hyper-sympathetic activity was manifested by shortened RR-interval shortened, increased LF, HF and LF/HF ratio and elevated plasma norepinephrine levels after LSG stimulation. AF inducibility was facilitated and atrial electrophysiological changes (shortening of ERP, increasing of ERP dispersion) was aggravated by hyper-sympathetic nerve activity.

The following electrophysiological mechanisms have been proposed to account for AF: sympathetic stimulation shortens action potential duration (APD) and ERP, increases ERP dispersion, induces early after depolarisations (EADs), decreases wavelet re-entry and increases spatial heterogeneity of atrial electrical activity.

### Catheter-based Renal Sympathetic Denervation as a Potential Strategy for AF Management

The sophisticated network of afferent and efferent sensory, chemo- and baroreceptor nerve fibers is distributed as a network in the adventitia of the renal artery and throughout the kidney [28]. The sympathetic nerves to the kidneys terminate in the blood vessels, the juxtaglomerular apparatus, and the renal tubules [29]. Altering the signals from the kidney to the hypothalamus is expected to impact peripherally, including on arterial resistance, peripheral and central chemoreceptors, sympathetic activity of the kidney and the heart itself [30]. Recently, therapeutic catheter-based RSD has been applied in a controlled randomized trial for the treatment of resistant hypertension with a significant reduction of systolic blood pressure, diastolic pressure [7]–[10]. RSD was also shown to reduce resting HR in patients with resistant hypertension [31]. The reduction of central sympathetic stimulation associated with nerve stimulation from the carotid sinus may result in substantially reduced renal sympathetic efferent signaling [32]. In our study, we found that no significant difference in SBP was found after stimulation between the control group and RSD group and RSD decreased SBP after ablation. RSD significantly reversed the shortened RR-interval, increased LF, HF and LF/HF ratio and elevated plasma norepinephrine levels by LSG stimulation. These findings suggested that RSD significantly inhibited cardiac sympathetic activity. Therefore, our results confirmed the hypothesis that RSD can reduce not only local sympathetic activity (efferent renal sympathetic nerve activity and afferent renal nerve activity) but also whole-body sympathetic activities, including cardiac sympathetic activity.

RSD was attempted to prevent or terminate arrhythmia. In 2012, Ukena [33] et al. reported the first-in-man experience in two patients with chronic heart failure and electrical storm, in whom catheter-based renal sympathetic denervation was recommended and performed after other treatment options failed. They suggest that RDN is feasible even in cardiac unstable patients. Linz et al [11] first reported that renal denervation reduced the inducibility of obstructive sleep apnea-associated AF. A pronounced AERP shortening induced by vagally mediated negative tracheal pressure was modulated by RSD or atenolol. Recently, their study showed that electrical baroreflex stimulation significantly shortened atrial refractoriness, thereby causing increased AF inducibility. In contrast, RSD did not significantly modulate atrial refractoriness or AFinducibility [12]. Zhao et al [13] demonstrated that episodes and duration of AF could be reduced by RSD during 7-hour RAP in dogs. The reduction of AF inducibility might be associated with decreased activity of renin-angiotensin-aldosterone system. Notably, AF in all the three studies associates with a substantially increase in vagal activity, leading to inconsistent effects of RSD. It is not clear if the inhibitory effect of RSD on AF is related to inhibition of the excitatory reflexs of vagal activity.

In the present study, RSD significantly reduced AF inducibility and reversed the atrial electrophysiological changes.The results strongly suggested that RSD had a potent inhibitory effect on AF induced by hyper-sympathetic activity. The mechanism may be that the excitatory reflexes on central sympathetic outflow including cardiac sympathetic activity was inhibited by afferent renal nerve denervation.

The ventricular response rate in atrial fibrillation is often a manifestation of sympathetic state. We found that the shortened RR-interval (elevated ventricular rate) induced by LSG stimulation can be prolonged by RSD. The ventricular rate in AF has been observed to fall following renal denervation in resistant hypertension (Felix Mahfoud, personal communication). While this may represent changes in myocardial work and stress associated with blood pressure declines, and indicates reductions in direct cardiac sympathetic signaling [34]. Consistently, HRV analysis in this study showed that RSD markedly reversed the abnormity of the time and frequency domain parameters (SDNN, pNN50, LF, HF and LF/HF ratio) induced by hyper-sympathetic nerve activity. These results further demonstrated that RSD modulated the cardiac autonomic activity which innervated the atrium and facilitated AF.

### Study Limitations

Although we showed evidence that renal nerve stimulation or ablation could activate or suppress sympathetic activity by indirectly observe changes of HR and SBP, direct neural firing from the SG or renal sympathetic nerve was not recorded in this study. The spectral index LF, obtained from systolic arterial pressure variability (LFSAP) which can furnished additional information on the sympathetic vasomotor control and reflect sympathetic activity was not tested. Since renal arteriography was not performed before and after catheter-based renal sympathetic denervation in our study, whether the renal artery had obvious stenosis after ablation or not was unclear. In addition, anaesthesia is known to interfere with the cardiovascular autonomic control. Furthermore, we need to investigate long-term changes of AF induction, electrophysiological data, and concentrations of norepinephrine in the hypothalamic after RSD.

### Conclusions

Hyper-sympathetic activity may facilitate the initiation of AF and acute atrial electrophysiological changes. RSD significantly reduced AF inducibility and reversed the atrial electrophysiological changes induced by hyper-sympathetic activity. The mechanism maybe that excitatory reflexes on cardiac sympathetic outflow was inhibited by afferent renal nerve denervation.
