# Correction: Catheter-Based Renal Sympathetic Denervation Significantly Inhibits Atrial Fibrillation Induced by Electrical Stimulation of the Left Stellate Ganglion and Rapid Atrial Pacing

**DOI:** 10.1371/annotation/f5414425-512f-4832-9166-3a6132ccd068

**Published:** 2013-12-30

**Authors:** Yuemei Hou, Jialu Hu, Sunny S. Po, Huan Wang, Ling Zhang, Feng Zhang, Kun Wang, Qina Zhou

Affiliation number 1 for the first author is incorrect. The correct affiliation is: Department of Cardiovascular Diseases, The 6th People’s Hospital (Southern) affiliated to Shanghai Jiaotong University, Shanghai, China. 

